# Lattice Boltzmann Method of Different BGA Orientations on I-Type Dispensing Method

**DOI:** 10.1371/journal.pone.0159357

**Published:** 2016-07-25

**Authors:** Aizat Abas, Z. L. Gan, M. H. H. Ishak, M. Z. Abdullah, Soon Fuat Khor

**Affiliations:** 1 School of Mechanical Engineering, Universiti Sains Malaysia, Engineering Campus, 14300 Nibong Tebal, Penang, Malaysia; 2 School of Aerospace Engineering, Universiti Sains Malaysia, Engineering Campus, 14300 Nibong Tebal, Penang, Malaysia; University of Catania, ITALY

## Abstract

This paper studies the three dimensional (3D) simulation of fluid flows through the ball grid array (BGA) to replicate the real underfill encapsulation process. The effect of different solder bump arrangements of BGA on the flow front, pressure and velocity of the fluid is investigated. The flow front, pressure and velocity for different time intervals are determined and analyzed for potential problems relating to solder bump damage. The simulation results from Lattice Boltzmann Method (LBM) code will be validated with experimental findings as well as the conventional Finite Volume Method (FVM) code to ensure highly accurate simulation setup. Based on the findings, good agreement can be seen between LBM and FVM simulations as well as the experimental observations. It was shown that only LBM is capable of capturing the micro-voids formation. This study also shows an increasing trend in fluid filling time for BGA with perimeter, middle empty and full orientations. The perimeter orientation has a higher pressure fluid at the middle region of BGA surface compared to middle empty and full orientation. This research would shed new light for a highly accurate simulation of encapsulation process using LBM and help to further increase the reliability of the package produced.

## Introduction

The earliest integrated circuits were packaged in ceramic flat packs that is then converted into dual in-line package (DIP). Due to limitations of DIP packaging for use on very-large-scale integration (VLSI) pin counts number, pin grid array (PGA) and leadless chip carrier (LCC) packages are introduced [1]. Historically, ball grid array (BGA) is introduced to overcome the limitations of the PGA technology. The conventional flat packages consist of very thin and closely spaced pins that are easily damaged and require close control of the soldering process.

BGA packages offer more advantages over PGA such as more interconnection pins rather than just perimeter, shorter lead that results in better performance at higher speeds and lower thermal resistance within silicon chip which allows more heat conducted out of the device faster [1]. BGA is attached to the printed circuit board (PCB) through a process called underfilling process [2,3]. Underfill process involves dispensing a controlled amount of material into a gap between chip and substrate and is found important in protecting and increasing reliability of the electronic packaging (EP). It can reduce the global thermal expansion, stresses and strain between the silicon chip and substrate. The gap between the chip and silicon has to be completely filled with underfilling mold in order to protect life of the chip assembly [4]. The interaction between underfill mold and solder bumps may induce possible void or air pocket formation and solder bump damaged due to improper selection of underfill material and processing parameter [2–5]. Therefore, a better understanding on the underfilling process is important and can be achieved through the virtual modeling technique [6,7].

Two main approaches can be considered in simulating the underfill flow propagation; either microscale or macroscale formulation. For the macroscale formulation, for instance finite volume method (FVM) or finite element method (FEM), the domain is discretized into volume or element that contains a collection of particles (macro scale) in which the physical properties (velocity, pressure and temperature) are represented by the nodal values [8–11]. Whereas in the microscale approach, the medium is assumed to be made up of particles that is continuously colliding with each other. Given that the microscale approach can be rather time consuming since each particle is tracked, a mesoscale method, namely lattice Boltzmann method (LBM), is introduced that considers a collection of particles as a unit [12–14]. The property of the particles collection in LBM is represented by a distribution function. Lattice Boltzmann Method (LBM) approach is actually developed from lattice gases or Bhatnagar-Gross-Krook (BGK) equation [12,15]. LBM considers a volume element of fluid that consist of collection of particles represented by particle velocity distribution function at each grid point. It considers particle distribution at lattice nodes rather than an individual particle. The fluid particles can collide with each other when moving and the moments of the particles population is calculated [16]. To recover the Navier-Stokes formulation from LBM formulation, Chapman-Enskog formulation is introduced to formulate LBM population equations in terms of its macro-scale counterpart. LBM describes the space dynamics by probability distributions that are 6-dimensional in phase space and this description of molecular level is larger than hydrodynamic level of Navier Stokes. This is because it is not subject to separation of time scale and is able to describe fluid with larger mean free paths. LBM can be used for solving incompressible, time-dependent Navier-Stokes equations and can be represented by complex physical phenomenon such as multiphase flow and chemical interactions between fluid and surrounding [8]. The best feature of LBM compared to other method is its ability to take into account the particle-particle interaction forces via the use of San-Chen interaction strength function, G at microscopic level for multi-phase simulation [17,18]. In addition, the volume of fluid (VOF) multiphase method can be used since it can accurately track flow front due to its ability to directly incorporate surface tension in its formulation [19].

Accurate and reliable prediction of the sources of error during package on package (POP) encapsulation process could increase the reliability of the package produced and in same time reduce the manufacturing cost. Currently, most of the researches on POP are mainly simulated using FVM based software in which is based on macroscale formulation. C.Y. Khor et al. (2011, 2013 and 2014) have conducted three different studies using ANSYS on the ball grid array (BGA) package encapsulation process by considering three different parameters which is inlet pressure, silicon chip thickness and solder bump arrangement [4,20,21]. C.Y. Khor et al. (2011) highlights the importance of inlet pressure parameter on FSI of BGA package encapsulation. The FSI analysis is conducted by using Mesh-based parallel Code Coupling Interface (MpCCI) method with finite volume coding (FLUENT) and finite element coding (ABAQUS). They found out that the deformation and stress of the die increases exponentially with the increase of the inlet pressure, and the maximum stress on the solder bump is concentrated near inlet gate. An inlet pressure of 0.2 MPa will cause the fluctuation phenomena on the silicon die that is subsequently lead to unstable flow and incomplete filling in the encapsulation process. Therefore, they suggested that the inlet pressure should be controlled below 0.175 MPa to prevent possible occurring of fluctuation phenomena during the encapsulation process. C.Y Khor et al. continued their research by considering the influence of silicon chip thickness on FSI of BGA package encapsulation [22]. The same MpCCI method with FLUENT and ABAQUS is used and they discover that the thickness of the chip has an exponential trend to the maximum deformation and stress. The thin chip will produce the greatest deformation and stress concentration on the silicon chip and solder bump. The downward deformation of the middle chip region will decrease the gap height and increase the resistance to the flow. This space will then be filled with voids beneath the deformed chip leading to issues with “pop corning” effect. C.Y Khor et al. also conducted a study regarding the influence of solder bump arrangements on FSI of BGA package encapsulation [21]. They concluded that the full array solder bump array package encounters lower stress and deformation during encapsulation. The deformation was found around the region without the solder bump which is located closer to the inlet gate (C1, C2, C4 and C5) and at the center of the chip (C3). The deformation and stress of silicon chip in C3 for the perimeter arrangement is the highest among all arrangements during the encapsulation process. However, highest percentage of void was found in C2 for the perimeter arrangement due to deformation at the center region and the crowding of solder bumps [4,23]. To the best knowledge of the authors, limited publications have been found utilizing Lattice Boltzmann method to simulate problem relating to BGA encapsulation process. However, studies on flow over obstacles relating to the current encapsulation problem have been conducted by various researches.

LBM has been successfully implemented in the simulation of immiscible binary mixture, as shown in the paper by E Orlandini et al. and MR Swift et al. Using collision rules based on the Cahn-Hillard approach which leads to a thermodynamically consistent equilibrium state, three distinct dynamical regimes are observed by E Orlandini, namely above, at and below the critical temperature. The study shows good agreement between simulation results and theoretical predictions for a wide range of parameters [24]. MR Swift et al. describe a lattice Boltzmann scheme which is capable of obtaining an isothermal model of phase separation that can correctly formulate the bulk and the interfacial dynamics at low temperatures [25]. MR Swift et al. later extended his study on the lattice Boltzmann scheme and successfully describe a lattice Boltzmann scheme for the simulation of phase separation in one- and two- dimensional flow. The results are shown to in good agreement with the analytical calculations.

For multiphase flow with large density ratios, HW Zheng et al. constructed a lattice Boltzmann model that gave better calculation efficiency compared with the previous schemes. The model itself shows good agreement with several numerical results [26]. Bo Yan and Guangwu Yan then proposed a steady-state lattice Boltzmann equation that is independent of time for steady incompressible flow by modifying the traditional LBE. The equation was found appropriate for simulating steady state Navier-Stokes equation [27].

In a separate study, using Shan-Chen multicomponent model, S Schmieschek and J Harting conducted studies on the determination of contact angle of droplets on hydrophobic surfaces. The dependence of the contact angle on the simulation parameters is investigated and different approaches to determine the contact angles are quantitatively compared. The result show that the method is capable of modelling the whole range of contact angle [28]. SV Lischuck et al. presented an algorithm based on a modified Gunstensen and Rothmann method to model a continuum surface tension generating algorithm for a two component lattice Boltzmann scheme. The suggested method shows significant improvement reduction the spurious velocities as reported in previous schemes [29,30].

With the growing trend that the electronic industry moving towards smaller sized package of up to nanoscale, the interparticle interaction became dominant and cannot be overlooked. To complicate matter, the fluid flow will interact directly with the solder balls resulting solder breakage and voids formation. In light of this problem, an accurate simulation that is able predict the sources of error relating to interaction between mold and BGA up to microscale level is required. Using LBM would help to uplift this issue given its ability to recover both formulation in macro- and micro-scale level (meso-scale). This would allow BGA developer to properly place the BGA with appropriate dimension; i.e. pitch and diameter of BGA.

In this paper, the bounce-back formulation is used during the mold flow formulation to account for the one way fluid-structure interaction. The free surface formulation will be used to track the movement of the mold during the encapsulation process. Volume of fluid (VOF) forced flow formulation is then applied to formulate the multi-phase flow regime of the mold and air regions. The free surface region will be tracked as the movement of the flow front comes into contact with the solder balls.

## Materials and Methods

### Problem description

**Fig 1:**
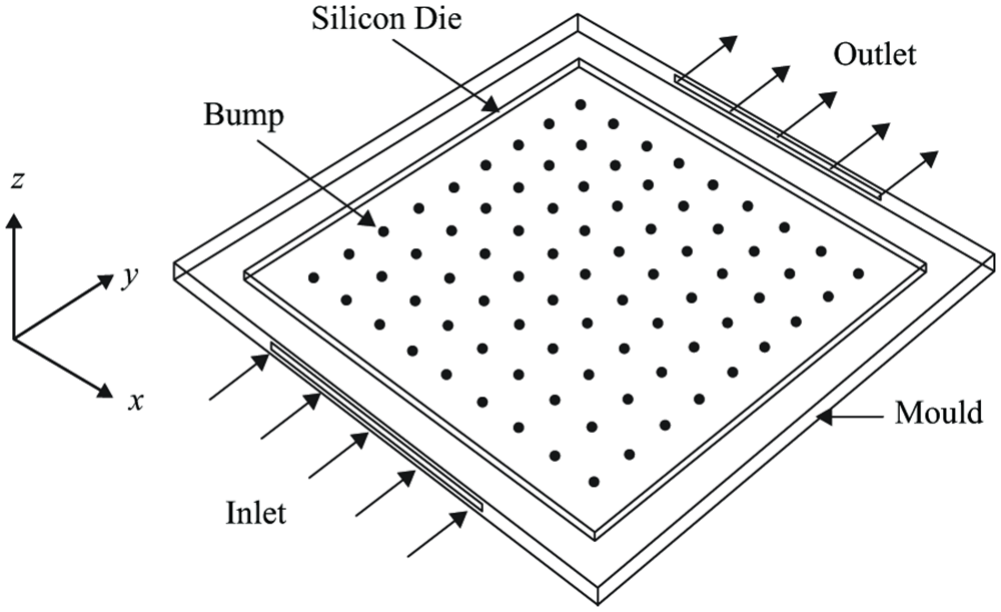
Schematic diagram of flip-chip boundary conditions.

Electronic packaging (EP) is an enclosure and protective features for the electronic products. The process of fabrication, assembly and testing of EP may involve a lot of complex interactions with physical phenomena such as temperature, fluid flow, electromagnetic and stress. This project is related to 3D fluid flow simulation through a ball grid array (BGA). Fig 1 depicts the schematic diagram of the underfilling process for the flow. The BGA consists of square array of spherical balls across the surface attached to another piece of printed circuit board (PCB). The underfilling process will be simulated using Palabos software that is based on LBM formulation.

**Fig 2:**
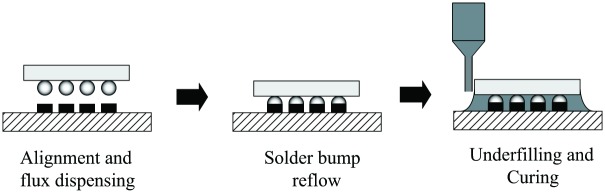
Underfilling Process Flow.

Fig 2 shows the capillary underfill process involving the dispensing process of controlling the amount of material into the gap between the chip and substrate. The underfill encapsulation of BGA will provide protection to increase reliability of the electronic package as it can reduce the global thermal expansion, stresses and strains between the silicon chip and substrate. The gap between the chip and silicon has to be completely filled with underfill material (underfill material flow correctly) in order to protect the life of the chip assembly [16]. Common issue relating incomplete filling may lead to voids formation.

### Experimental setup

**Fig 3:**
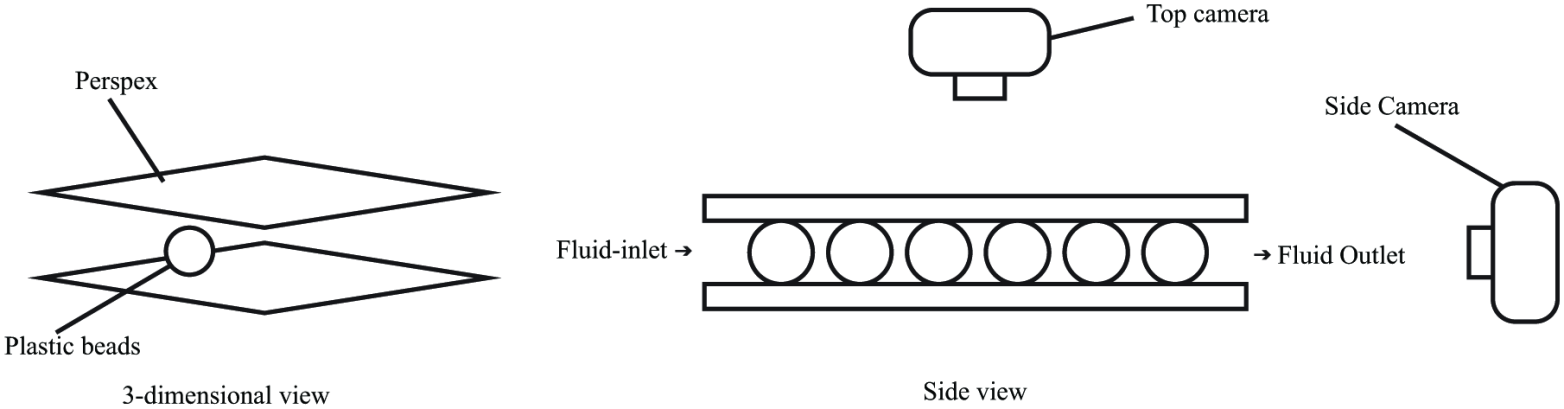
Experimental setup of BGA.

Scaled-up experiment is carried out by using plastic beads and transparent Perspex that is 4 times larger than the real ball grid array (BGA) size as it is highly complicated to conduct an experiment on a small scale setup. A test fluid with constant viscosity is also used as the EMC. The purpose of the scaled-up experiment is to validate the simulation result and the conformity of LBM based software, Palabos, in simulating the fluid flows. The experiment is conducted based on the following steps:

1. First, cut the Perspex according to the scaled-up dimension and the plastic beads are stick on the Perspex surface with 3 different types of orientation (perimeter, middle empty and full). The experimental setup of BGA is illustrated in Fig 3.
2. As shown in Fig 3, two cameras are set up on two different positions where the main camera captures the fluid flow from the top view while the secondary camera captures the fluid flow from the side view. The fluid flows for the experiment is in I-type flow in which the fluid flow enters at one end and exits from the opposite end with the other two sides closed.
3. Both cameras will capture the fluid flow slightly before the start of the injection of fluid into the space of BGA model in order to ensure the video is recorded completely during the whole process.
4. The fluid is constantly injected through the I-line gap on the perimeter BGA model from the inlet until fluid flow completely covers the whole surface of the underfill. The setup is then cleaned and dried to ensure same conditions are applied on all repeated experiments.
5. The experiment is repeated for different orientation of BGA (middle empty and full array). The same fluid injection and video recording step are carried out for the subsequent experiments.

### Governing equations

The results shown in this section are formulated using D3Q19 lattice model. The LBM equation can be summarized in Eq (1):

f(r+cdt,c+Fdt, t+dt)−f(r,c,t)=Ω[f(r,c,t)] (1)

in which the left hand side represents the streaming step. The right hand term denotes the collision term that can be represented using the well-known Bhatnagar-Groos-Krook (BGK) model as given in Eq (2).

$$\Omega =\omega \left(f^{eq}-f\right)=\frac{1}{\tau }\left(f^{eq}-f\right)$$ (2)

*ω* and *τ* denote the relaxation frequency and time. *f*^*eq*^ represents the equilibrium function that relates to the lattice arrangement.

**Fig 4:**
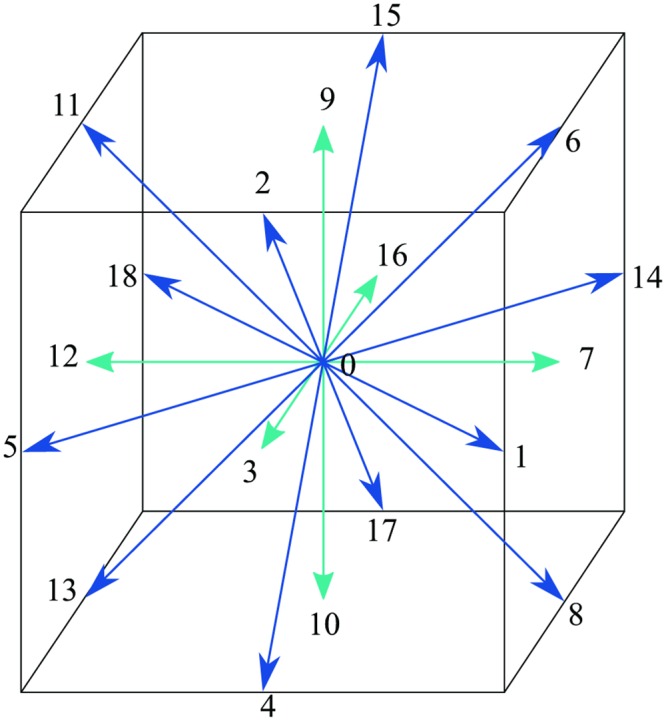
3D Lattice arrangements for D3Q19.

The equilibrium function, *f*^*eq*^, can be described as:

$$f^{eq}\left(\rho ,u\right)=\rho w\left[1+\frac{1}{c_{s}^{2}}\left(c.u\right)+\frac{1}{2c_{s}^{4}}{\left(c.u\right)}^{2}-\frac{1}{2c_{s}^{2}}\left(u.u\right)\right]$$ (3)

in which *w* represents weighting function across different lattice links. For the case of D3Q19 lattice model as depicted in Fig 4, the weighting functions can be described in Table 1 as:

**Table 1:** Weighting functions for D3Q19.

Model	$c_{s}^{2}$	Node no.	Weight
D3Q19	1/3	*f*_0_	1/3
		*f*_1_−*f*_6_	1/18
		*f*_7_−*f*_18_	1/36

Microscopic velocities for a D3Q19 lattice model is given as:

$$\begin{array}{c} e_{0}=\left(0,0,0\right)\\ e_{1,2}=\left(\pm 1,0,0\right)\\ e_{3,4}=\left(0,\pm 1,0\right)\\ e_{5,6}=\left(0,0,\pm 1\right)\\ e_{7-10}=\left(\pm 1,\pm 1,0\right)\\ e_{11-14}=\left(\pm 1,0,\pm 1\right)\\ e_{15-18}=\left(\pm 1,0,\pm 1\right) \end{array}$$ (4)

#### Conventional BGK collision model

The underlying theory of LBM is based on the discrete Boltzmann equation. Due to the complicated collision term that exist in the right hand side of the Boltzmann equation, it is difficult and burdensome to solve. To ease the computation effort, Bhatnagar, Gross and Krook (BGK) proposed a simplified version of the collision operator [31]. The collision operator can be replaced as

$$\Omega =\omega \left(f^{eq}-f\right)=\frac{1}{\tau }\left(f^{eq}-f\right)$$ (5)

where $\omega \;=\;\frac{1}{\tau }.$ The coefficient *ω* denotes the collision frequency and *τ* is the relaxation factor. *f*^*eq*^ is the Maxwell Boltzmann equilibrium distribution function. By substituting the approximate collision operator, the discrete Boltzmann equation can be shown as

$$f_{i}\left(\vec{x}+{\vec{e}}_{i}\;,\;t+1\right)=f_{i}\left(\vec{x},t\right)+\Omega$$ (6)

where *i* is the index selected between the possible discrete velocity directions and *e*_*i*_ is the direction of the selected velocity.

The fluid density and macroscopic velocity can be found from the moment of the distribution function as below

$$\rho =\sum_{i}f_{i}$$ (7)

$$\vec{u}=\frac{1}{\rho }\sum_{i}f_{i}{\vec{e}}_{i}$$ (8)

Subsequently, the equilibrium distribution, *f*^*eq*^, can be arranged according to the Maxwell-Boltzmann distribution form as

$$f_{i}^{eq}\left(\vec{x},t\right)=w_{i}\rho \left[1+3\left(\vec{e_{i}}\vec{u}\right)+\frac{9}{2}{\left(\vec{e_{i}}\vec{u}\right)}^{2}-\frac{3}{2}{\vec{u}}^{2}\right]$$ (9)

Eq 5 will replace the commonly used Navier-Stokes equation in CFD simulations. It is also possible to derive N-S equation from Boltzmann equation using the Chapman-Enskog model.

#### LBM Free-surface multiphase formulation

The volume of fraction *ϵ* based on the volume-of-fluid (VOF) formulation is introduced to the current LBM setup to distinguish the fluid (mold) and gas domain regions [19]. The VOF value of *ϵ* for each of the fluid and gas domain can be specified in the range between value of 0 and 1. The fluid mass content in a domain is calculated according to the volume of fraction *ϵ* and the density *ρ* as

M=ρ·ϵ (10)

As the fluid flow during the advection (stream step), dynamic mass tracking technique is implemented to track the gas, fluid and interface and is given by

$$\Delta m_{i}\left(x\right)=\{\begin{array}{cc} \;0, & \;if\;gas\;at\;r+c\;dt\\ \;f_{i}\left(r+c\;dt\right)-f_{i}\left(r\right), & \;if\;liquid\;at\;r+c\;dt\\ \frac{1}{2}\left[\varphi \left(r\right)+\varphi \left(r+c\;dt\right)\right]\left[f_{i}\left(r+c\;dt\right)-f_{i}\left(r\right)\right], & if\;interface\;at\;r+c\;dt \end{array}$$ (11)

At the interface, the distribution function must take in to account the neighbouring gas cells as

$$f_{i}\left(r-c\;dt\right)=f_{eq,i}\left(\rho_{g},u\left(r\right)\right)-f_{i}\left(r\right)$$ (12)

in which *ρ*_*G*_ is related to the gas pressure and the lattice speed of sound *c*_*s*_ by

$$\rho_{G}=\frac{1}{c_{s}^{2}}P_{G}$$ (13)

To take into account the effect of surface tension during the mold flow propagation, the value of *ρ*_*G*_ is modified as

$$\rho_{G}=\frac{1}{c_{s}^{2}}\left(P_{G}+2\sigma \kappa \left(r,t\right)\right)$$ (14)

in which *σ* is the surface tension of the liquid, and *κ*(*r*, *t*) is the local curvature of the free surface. The surface tension is then calculated according to the following formulation

$$\sigma =\frac{\rho gL^{2}}{Bo}$$ (15)

with *B*_*o*_ representing the Bond number, *g* the gravity and *L* is the lattice size. The local curvature points are the calculated using the marching cure algorithm to reconstruct the mold front free-surface [32].

### Numerical simulation

**Fig 5:**
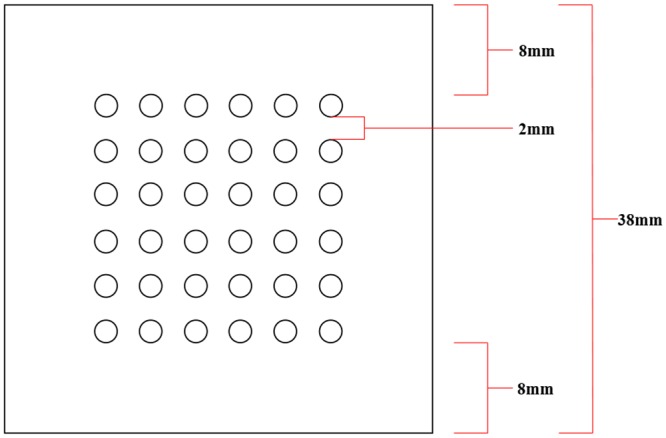
Solder bump arrangement dimension on simulation model (Full orientation).

A 3D ball grid array (BGA) package model was created with a scaled-up dimension of four times the real industrial BGA dimensions. Solder bump of diameter of 2mm is placed on top of the surface of BGA with 3 different types of solder bump orientations namely perimeter, middle empty and full orientations. The solder bump counts for these three orientations is as stated in Table 2. The inlet gate has a dimension of 38mm × 2mm whereas the outlet gate has the same dimension but located at the opposite side. The dimension of solder bump arrangement is illustrated in the Fig 5. The space between each of the solder bump is 2mm and the distance of solder bump from the edge of BGA is 8mm. In this simulation, the fluid flows via capillary action without the initial velocities specified. Capillary action is the ability of fluid flow through narrow spaces without the aid of external force, i.e. pressurized flow. The parameters used to model capillary action utilizes the combination of surface tension and adhesive forces between the fluid and container surface.

**Table 2:** Solder bump counts in different solder bump arrangements/ orientations.

Orientation	Perimeter	Middle Empty	Full
Solder bump number	20	32	36

**Fig 6:**
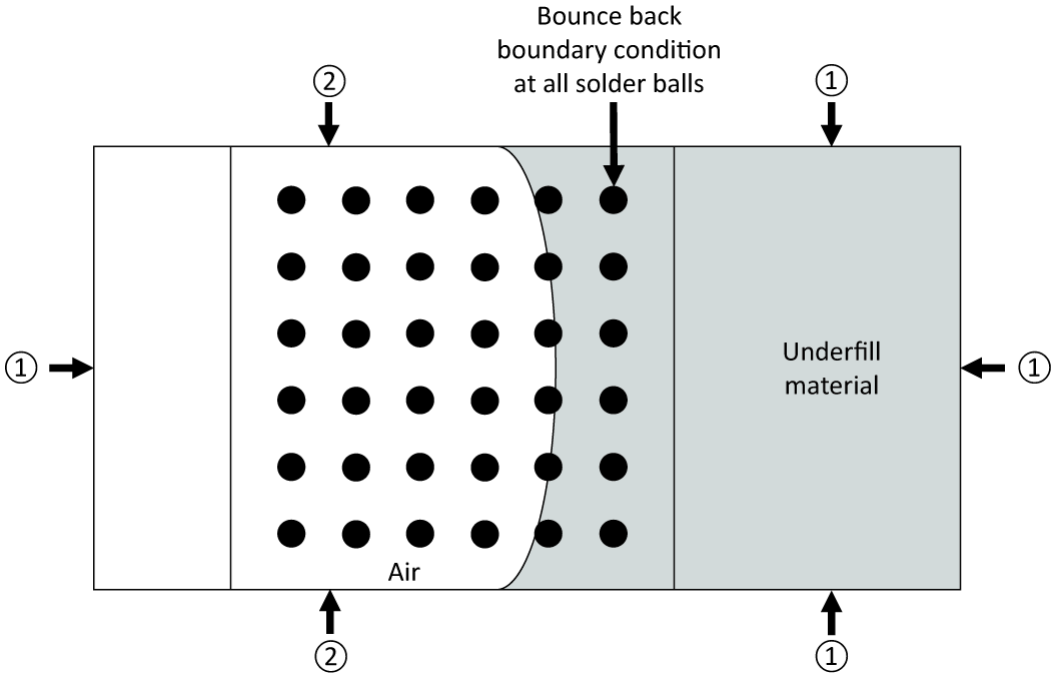
LBM boundary conditions setup for periodic and bounce back conditions (①—Periodic boundary conditions, ②—Wall boundary conditions).

The boundary conditions of the numerical model is defined as shown in Fig 6. Periodic boundary conditions are imposed in the right half of the channel at top and bottom surfaces in order to mimic an “infinite reservoir”. Periodic boundary conditions are also imposed at the two lateral sides such to ensure total mass conservation inside the system. At the solid surface, bounce back boundary conditions for the particle distributions are applied.

The encapsulant material used is the epoxy-molded compound (EMC) with properties summarized in Table 3.

**Table 3:** EMC encapsulant material properties.

Parameter	Value
Density (kg/m^3^)	1042
Dynamics viscosity (Pa·s)	2.2
Kinematic viscosity (mm^2^/s)	2111.3
Surface tension (N/m)	0.047
Bond number	1108

The density of the underfill fluid that is used in this simulation is 1042 kg/m^3^ with dynamic viscosity, μ of 2.2Pa∙s as used previously in the underfill encapsulation process [33,34]. The bond number used is 1108 and is calculated using the following formulation:

$$Bo=\frac{\rho gL^{2}}{\sigma }=\frac{1024\;\left(9.81\right){\left(0.072\right)}^{2}}{0.047}=1108$$

The contact angle used to measure contact between wetting (mold epoxy) and non-wetting fluid (air) is set at 80°. The number of D3Q19 lattices on the z-direction of this simulation model is 60 and the time difference, ΔT, of each simulation interval is set at 0.001 s.

#### Capillary underfill formulation

**Fig 7:**
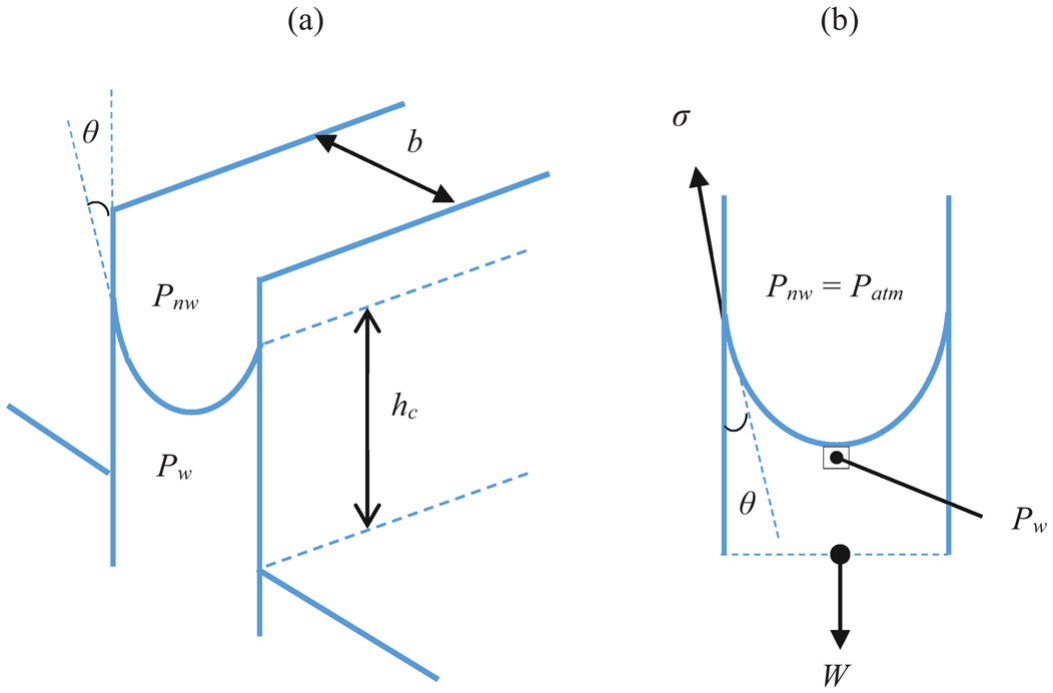
Capillary between two parallel plates: (a) The schematic diagram and (b) free body diagram.

In capillary underfill (CUF) encapsulation process, interaction occurs between two different fluids, the encapsulant and air, with the BGA surface. The capillary force is formulated to estimate the capillary flow advancements between two parallel plates as shown in Fig 7(a).

Fig 7(b) shows the free body diagram of the CUF flow. The computation of capillary force is derived according to the capillary rise formulation in Eqs (14) and (15).

$$\gamma_{f}h_{c}\frac{\pi b^{2}}{2}=\sigma \left(\pi b\right)cos\theta$$ (16)

$$h_{c}=\frac{2\sigma }{b\gamma_{f}}$$ (17)

The capillary pressure is defined by the pressure difference between the wetting and non-wetting fluids. The pressure of the non-wetting fluid, air in this case is equivalent to atmospheric pressure. It can be expressed as:

$$\Delta P_{c}=P_{nw}-P_{w}$$ (18)

$$\Delta P_{c}=\gamma_{f}h_{c}$$ (19)

$$\Delta P_{c}=\frac{2\sigma }{b}$$ (20)

where *γ*_*f*_ is the unit weight of fluid, h_c_ is the capillary height, σ is the surface tension coefficient and b is the distance between two parallel plates.

Significant deduction can be made from the formulation, in which the capillary pressure is proportional to surface tension and inversely proportional to the gap height. This indicates that for two parallel plate with smaller gap height, larger capillary pressure would be required to drive the flow. Pressure is reduced by loss due to the existence of solder bumps.

$$\Delta P_{t}=\Delta P_{c}-\;\Delta P_{b}$$ (21)

The effect of solder bump should not be neglected, particularly when a high density of solder bumps are present. In finding the suitable BGA size, the gap height needs to be monitored since no generation of encapsulant can occur if the gap is small.

## Results and Discussion

**Fig 8:**
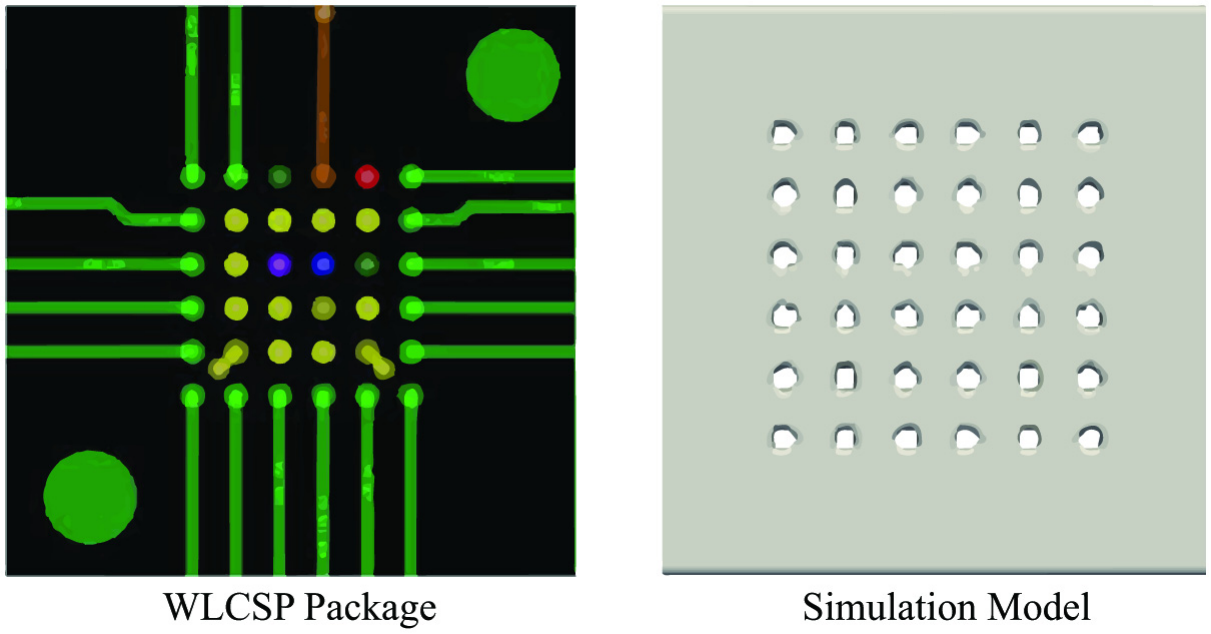
Real world WLCSP package [28] and its simulation model.

The simulation model in full orientation of this study consists of 36 of solder bumps in square array (6x6) that duplicates the model of 36 bump wafer level chip scale package (WLCSP), MXO3L-1300E-UWG36 and ICE5LP4K-SWG36 [28], as shown in Fig 8. Real industry test case is chosen to validate the capability of LBM in the simulation of the encapsulation process.

**Fig 9:**
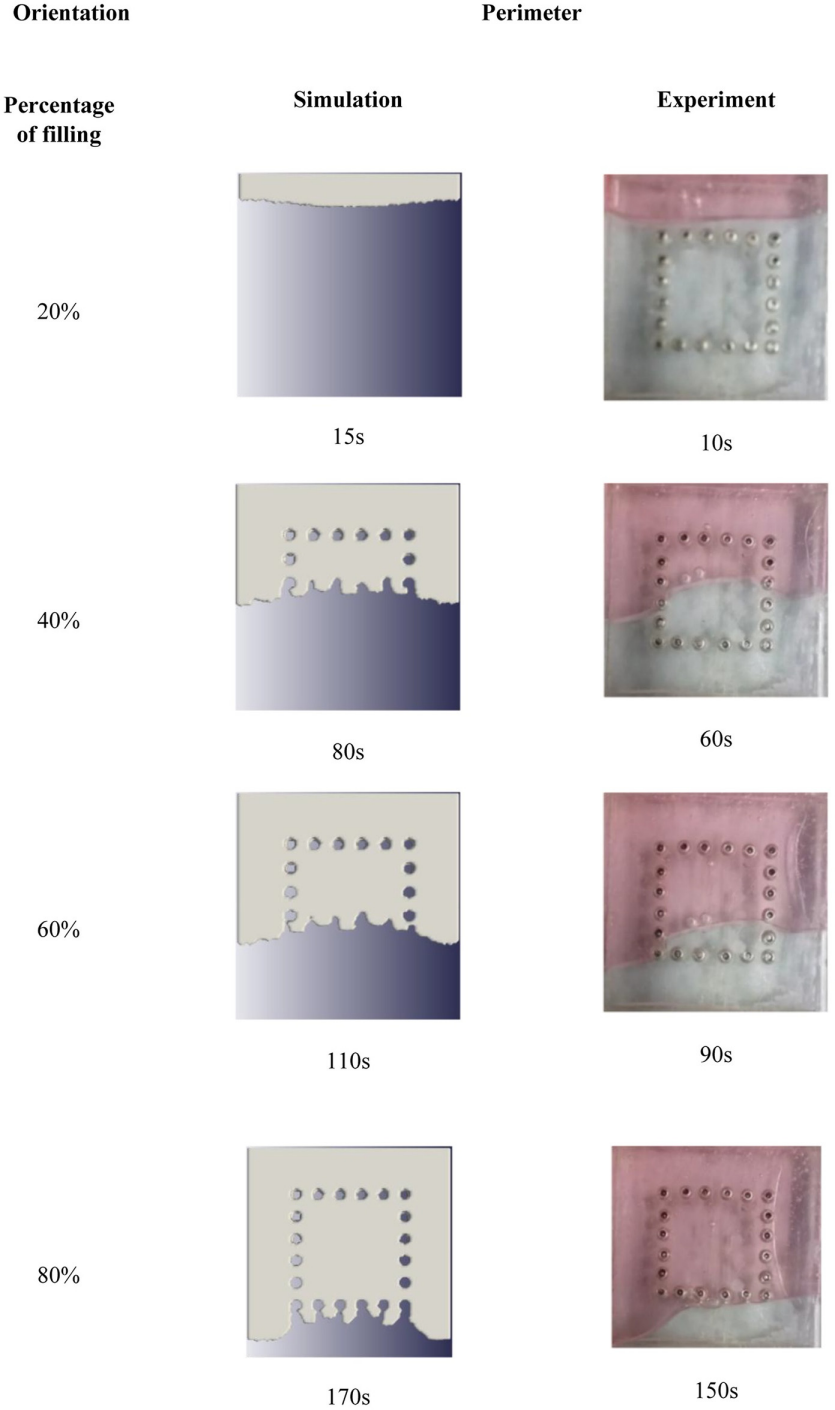
Comparison between simulation and experiment (Orientation—perimeter).

**Fig 10:**
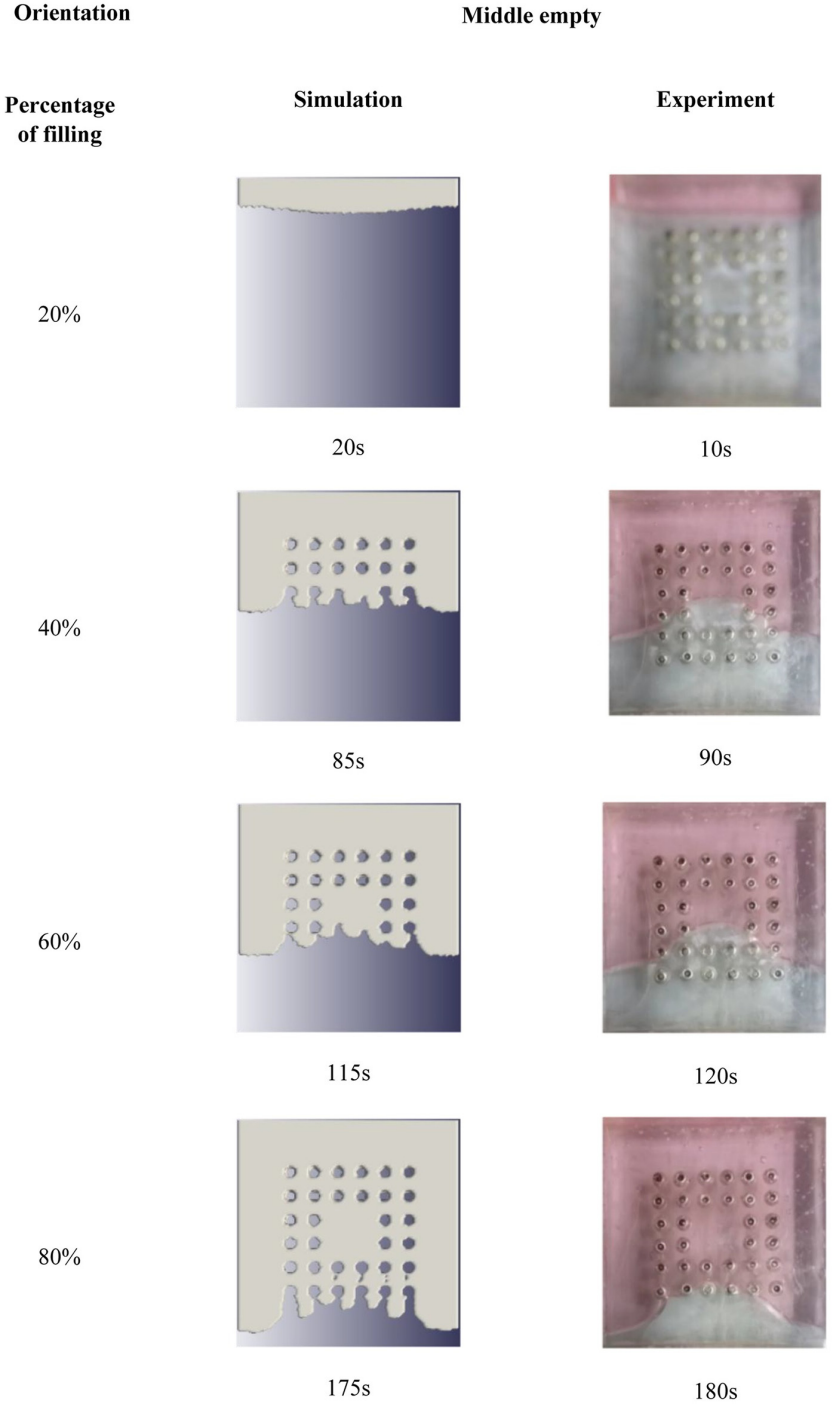
Comparison between simulation and experiment (Orientation—middle empty).

**Fig 11:**
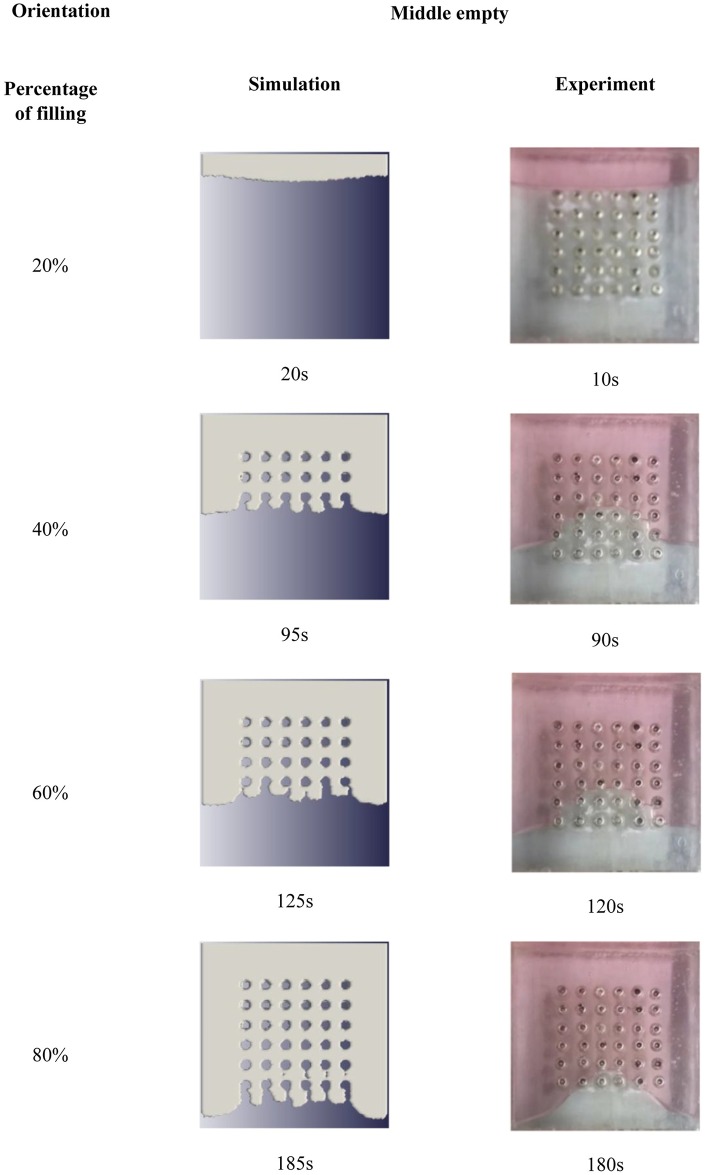
Comparison between simulation and experiment (Orientation—full).

**Fig 12:**
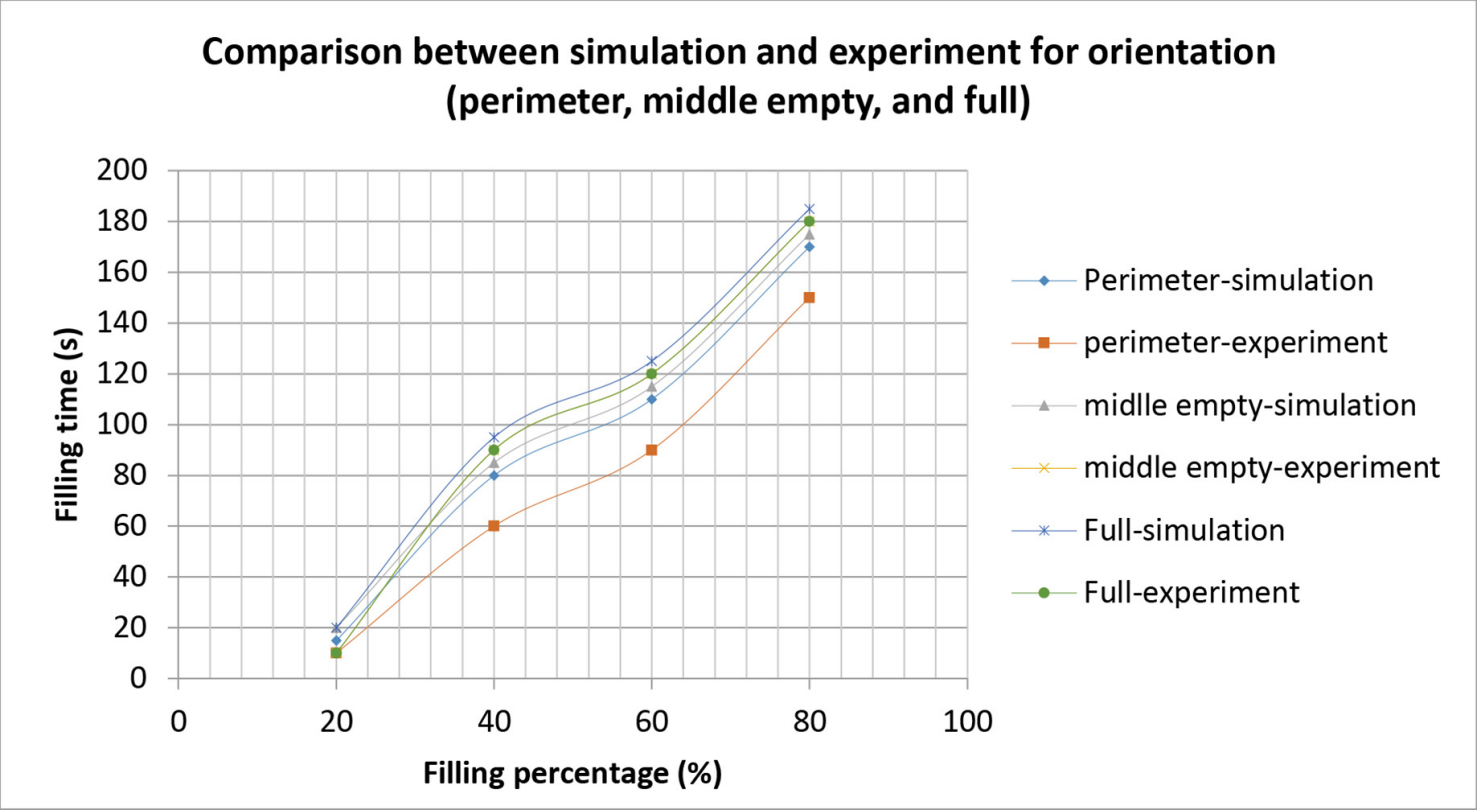
Graph of comparison of simulation and experiment for all orientations.

The simulation and experimental data are compared according to their filling percentage and filling time. Figs 9, 10 and 11 show the comparison between the simulation and experiment result based on filling percentage of 20%, 40%, 60% and 80%. The wettability obtained using LBM simulation might differ slightly since the formulation is based on micro-scale calculation which produces very high detail of the mold flow front propagation. As FVM is a macro-scale method, exact detail of the flow front might not be captured effectively as compared to LBM. The high level of detail produced in LBM simulation is the main reason why micro-defects can be detected with relative ease. Using conventional macro-scale formulation, for instance FVM and FEM, might produce similar wettability however the micro-sized defects might not be captured during the mold flow simulation. The wettability of the flow front can be describe according to the surface tension value. Higher surface tension corresponds to higher value of Bond number leading to an increase in the voids formation [35]. Table 4 consists of the times for specific filling percentage for all orientations. Based on the data from Table 4, a graph is constructed to compare between simulation and experimental observations for all orientations as shown in Fig 12.

**Table 4:** Data from comparison between simulation and experiment (perimeter, middle empty and full).

Orientation	Perimeter		Middle Empty		Full	
Filling Percentage (%)	Simulation	Exp	Simulation	Exp	Simulation	Exp
20	15s	10s	20s	10s	20s	10s
40	80s	60s	85s	90s	95s	90s
60	110s	90s	115s	120s	125s	120s
80	170s	150s	175s	180s	185s	180s

From Figs 9, 10 and 11, the result of simulation and experiment for middle empty and full orientation show good conformity in the flow front displacement whereas for perimeter orientation show slight discrepancy in terms of the simulated filling time lag. The conformance with experimental findings are shown by perimeter orientation with exact flow front shape observed compared to the experiment result. In comparison, the graph plotted in Fig 12 shows that the fluid filling time for the perimeter orientation is much faster compared to the middle empty and full orientations as the number of the solder bumps of perimeter orientation is the least. Do note that the curves for middle empty-experiment and full-experiment coincide each other as they exhibit similar results. The solder bumps surface act as an obstacle to hinder the flow. Therefore, the less number of the solder bumps indicates the faster the fluid flow across the BGA surface (faster underfilling process). In actual implementation, the use of perimeter orientation would involve faster package production, however, with relatively less solder ball connections, the use of middle empty and full orientations are still important.

**Fig 13:**
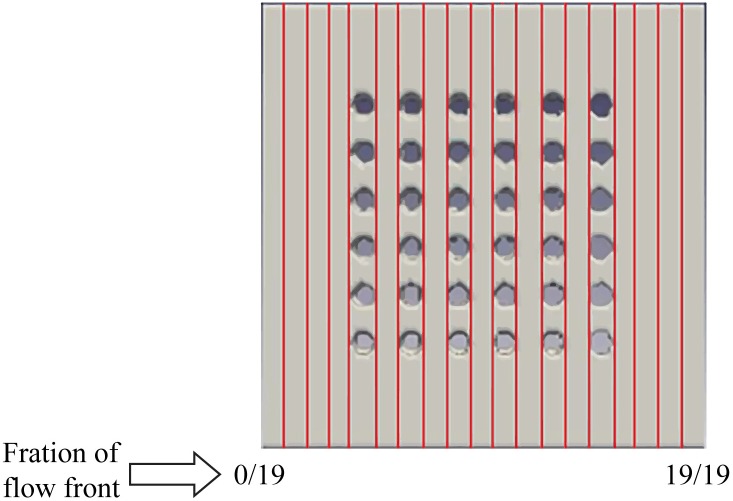
Width of ball grid array (BGA) surface (19 portions).

**Fig 14:**
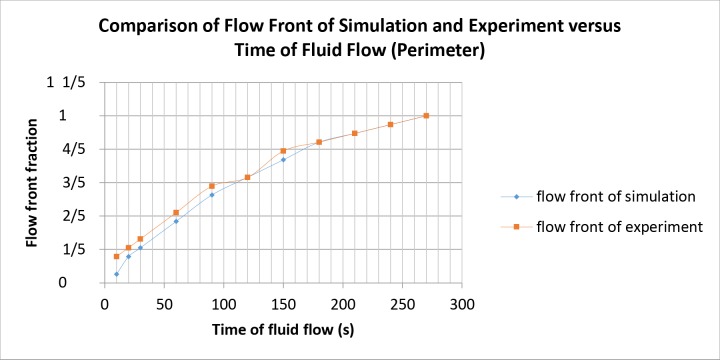
Comparison between simulation and experiment by filling time (perimeter).

**Fig 15:**
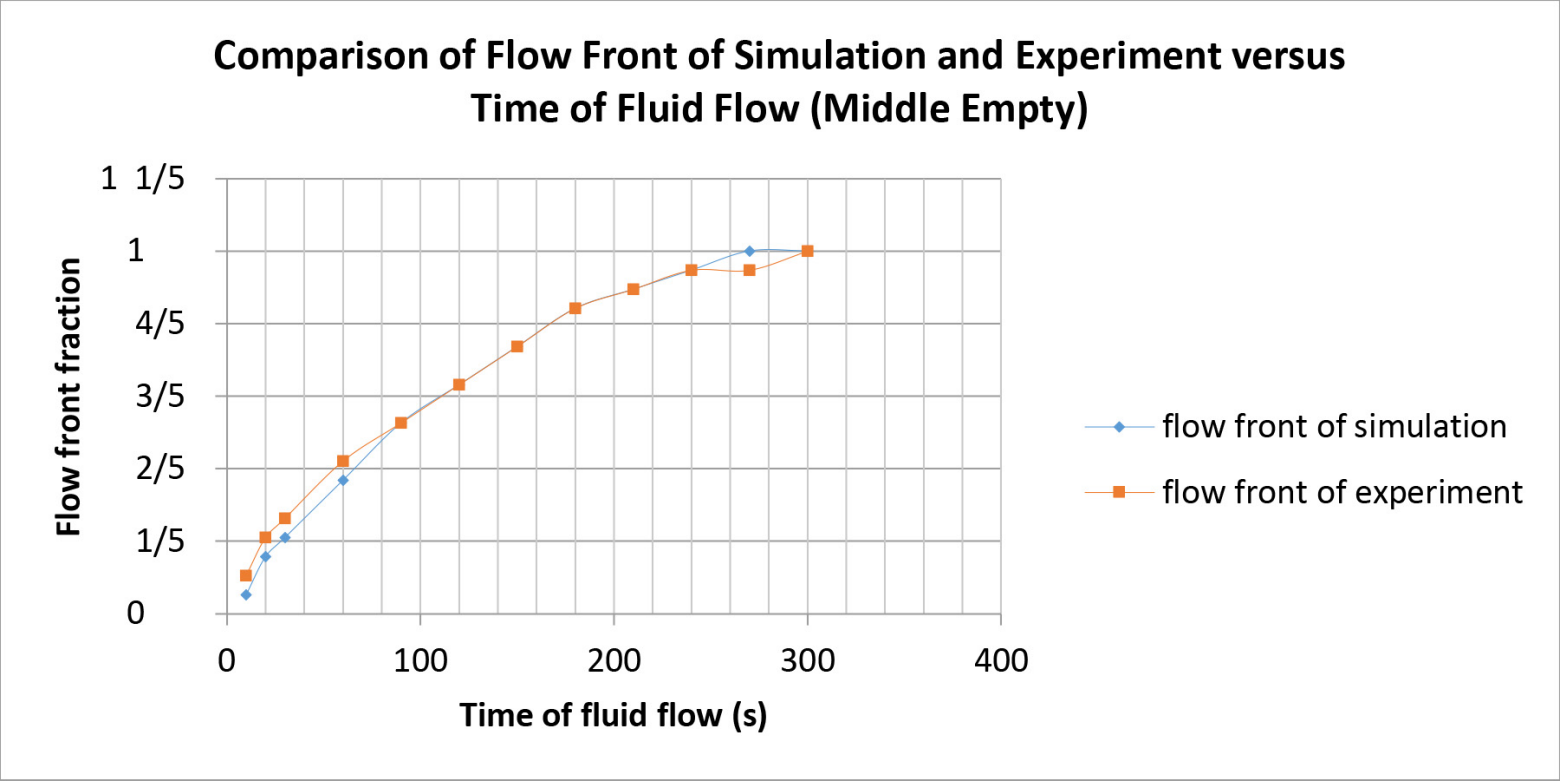
Comparison between simulation and experiment by filling time (middle empty).

**Fig 16:**
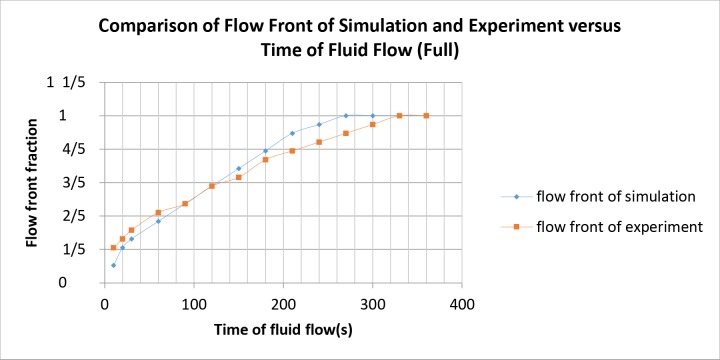
Comparison between simulation and experiment by filling time (full).

The flow front displacement in form these portions are determined for the filling time of 10s, 20s, 30s, 60s, 90s, 120s, 150s, 180s, 210s, 240s, 270s, 300s, 330s and 360s. The data of the flow front fraction displacement for each filling time interval is recorded in Table 5 and as depicted in Fig 13 respectively. For ease of comparison, Figs 14, 15 and 16 are plotted based on the data recorded in Table 5 for comparison of flow front fraction between simulation and experimental findings. It was found that the perimeter orientation has the fastest fluid filling time follower by middle empty and full orientations. The flow fronts for perimeter, middle empty and full orientations shows good side by side comparison between simulation and experimental data with slight discrepancy at the initial stage of the flow. The flow front however, tend to conform as the flow progresses. This discrepancy might be attributed to the slight human error in the application of the injected fluid at the beginning of the experiment which could have led to minimal increase of the flow rate at the early stage of the experiment. The overall flow fronts comparison between simulation and experiment have shown high conformity thereby proving the capability of LBM in simulating real world encapsulation problems.

**Table 5:** Flow front fraction comparison between simulation and experiment at different filling time.

Time (s)	Perimeter		Middle empty		Full	
	Simulation	Experiment	Simulation	Experiment	Simulation	Experiment
10	1/19	3/19	1/19	2/19	2/19	4/19
20	3/19	4/19	3/19	4/19	4/19	5/19
30	4/19	5/19	4/19	5/19	5/19	6/19
60	7/19	8/19	7/19	8/19	7/19	8/19
90	10/19	11/19	10/19	10/19	9/19	9/19
120	12/19	12/19	12/19	12/19	11/19	11/19
150	14/19	15/19	14/19	14/19	13/19	12/19
180	16/19	16/19	16/19	16/19	15/19	14/19
210	17/19	17/19	17/19	17/19	17/19	15/19
240	18/19	18/19	18/19	18/19	18/19	16/19
270	1	1	1	18/19	1	17/19
300	1	1	1	1	1	18/19
330	1	1	1	1	1	1
360	1	1	1	1	1	1

### Pressure distribution of fluid flow

**Fig 17:**
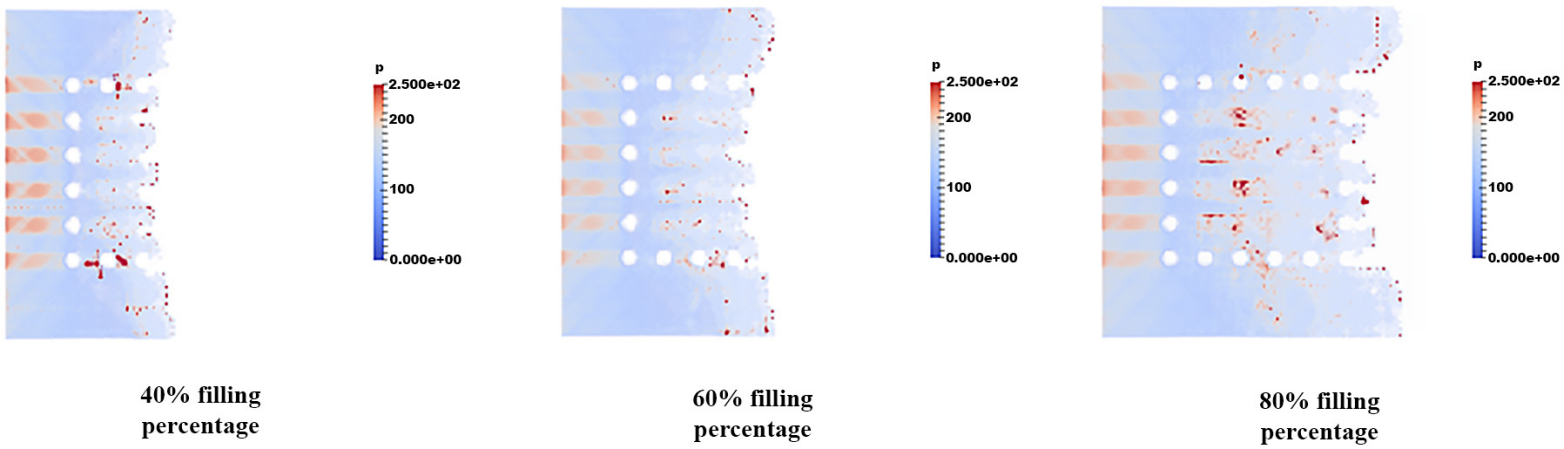
Pressure distribution of fluid flow (Orientation-perimeter).

**Fig 18:**
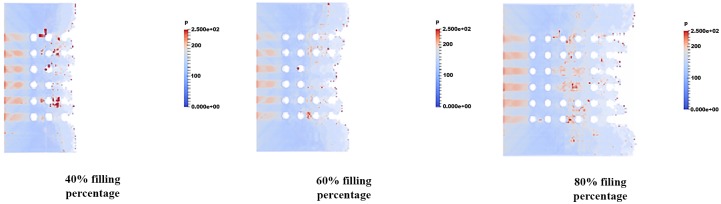
Pressure distribution of fluid flow (Orientation-middle empty).

**Fig 19:**
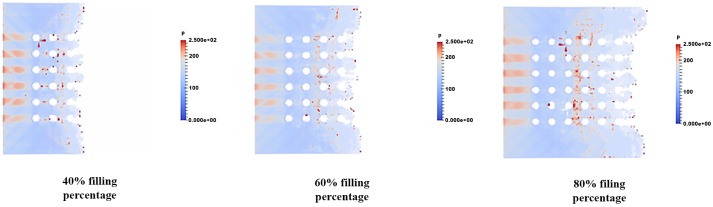
Pressure distribution of fluid flow (Orientation-full).

The pressure distribution is observed in filling percentage of 40%, 60% and 80% for all orientations are depicted in Figs 17, 18 and 19 respectively. Figs 17, 18 and 19 show the non-uniform pressure distribution throughout the surface of ball grid array (BGA) due to unbalance fluid flow. The flow front of fluid flow has a curved shape due to obstruction of solder bumps at the middle of BGA surface which result in the slower fluid flow in the middle than the edge of BGA. The unbalance fluid flow causes the uneven pressure distribution throughout the BGA surface. From the Figs 17, 18 and 19, perimeter orientation shows higher pressure at middle portion of BGA compared to the middle empty and full orientation due to the least number of solder bumps support at the middle portion of BGA with perimeter orientation. Less solder bumps in the middle portion of perimeter orientation causes the surface of the BGA to curve downward and reduce the gap fluid flows. Subsequently, this induces high pressure spot at the middle portion. The high pressure fluid flow may lead to unintended defects such as nearby solder bumps damaged and chip deformation. Incidentally, increasing the number of solder bumps reduce the high pressure spot of the fluid flow. The inlet region of the fluid flow also shows high pressure distribution for all 3 different orientations. This is partly due to reverse flow occurring at the contact surface of the front face of the solder bumps. Fig 17 shows the example of large pressure fluctuation during the encapsulation process. At 40% filling percentage, huge pressure variations are shown at the region near the second row. Similarly, these huge pressure variations are mainly caused by reverse flow issue due to continuous injection of the mold causing reduction in velocity of the flow front. This reduction in velocity leads to increase in pressure, resulting high pressure fluctuation at 40% filling. The pressure fluctuations however tend to depreciate as the filling percentage increases from 40% to 60%. For 80% filling percentage, similar pressure fluctuation is observed due to the flow front interaction with the final row of the solder bumps. For perimeter orientation as depicted in Fig 17, the region of high pressure still exists typically at the solder ball free region. This region could lead to package deformation due to pressure differential.

### Pressure distribution of fluid flow

**Fig 20:**
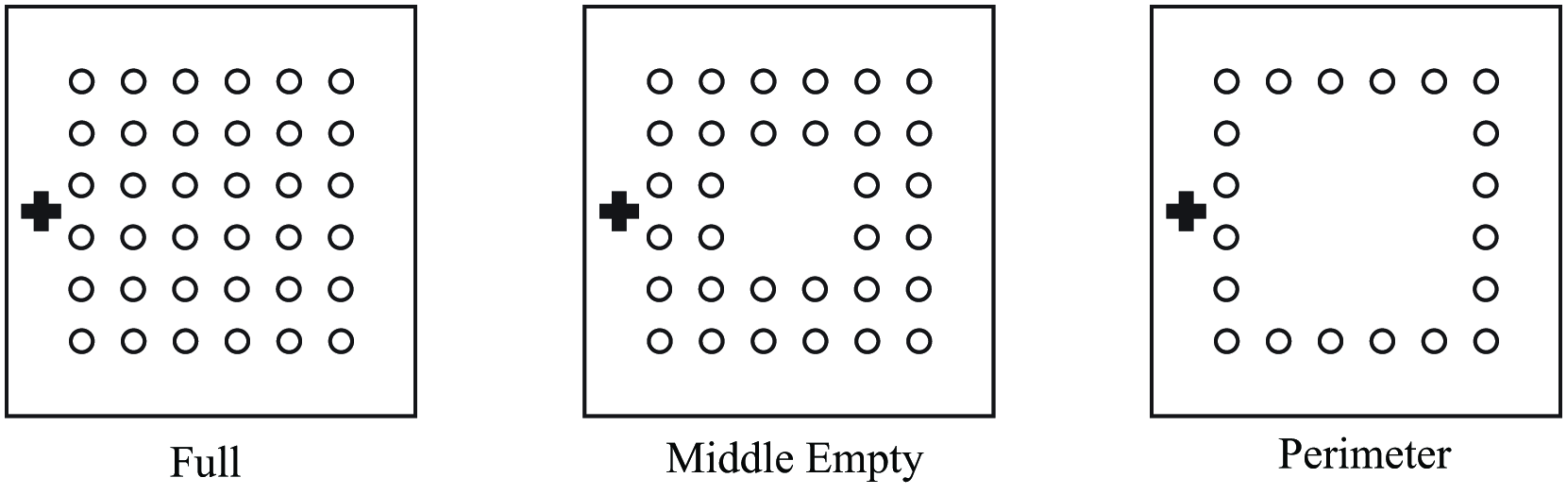
Position of Probe location in the simulation.

**Fig 21:**
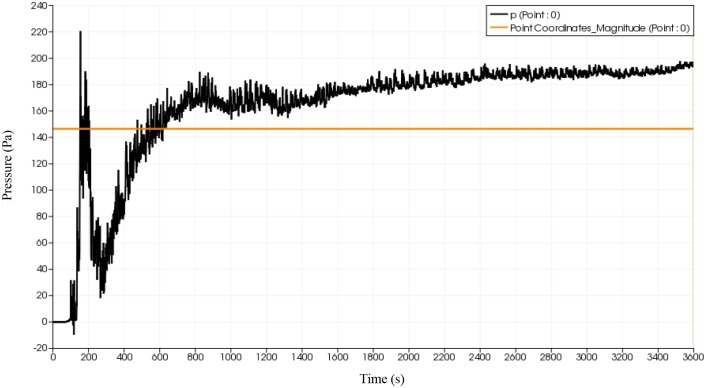
Graph of pressure over time (full) from simulation data.

**Fig 22:**
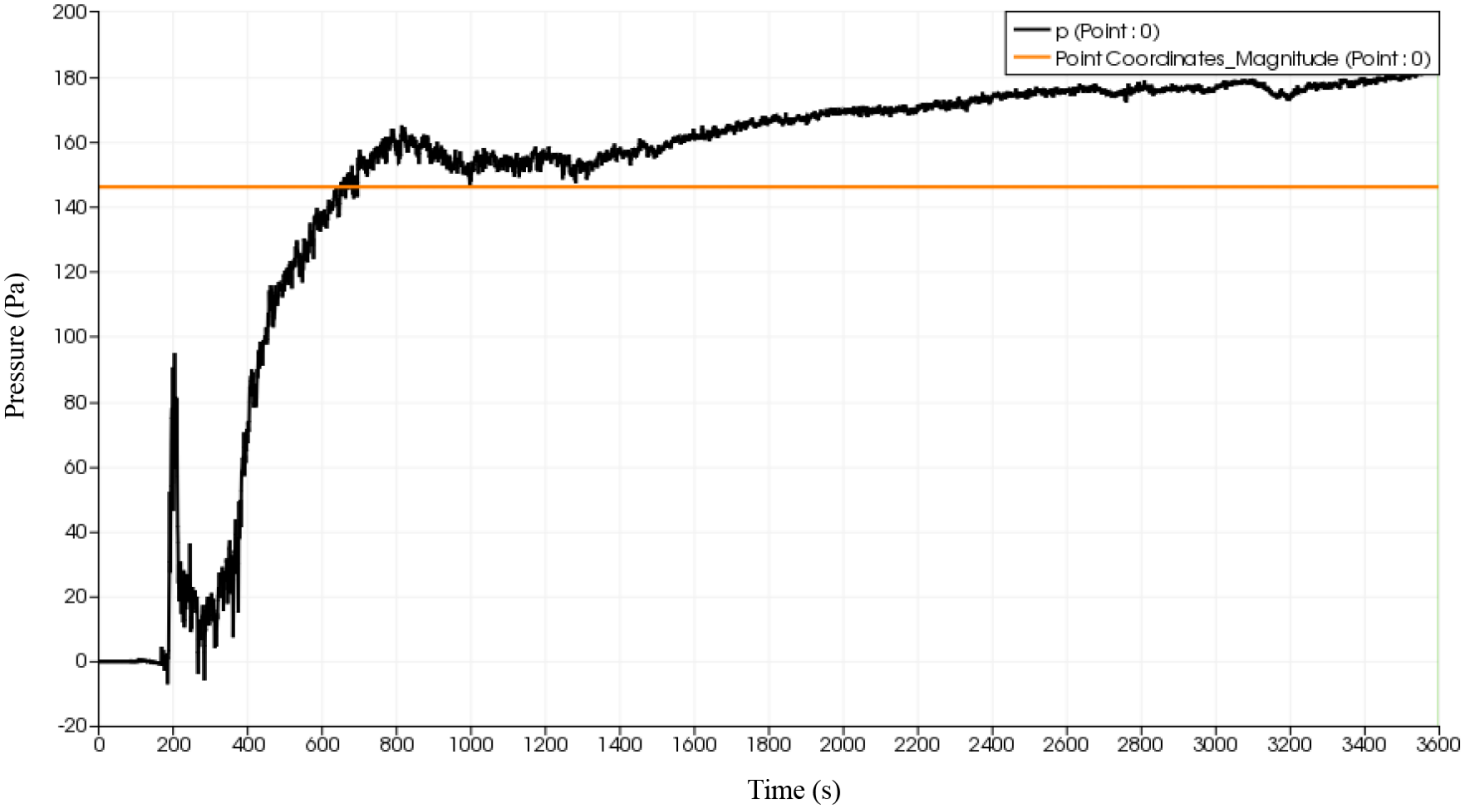
Graph of pressure versus time (middle empty) from simulation data.

**Fig 23:**
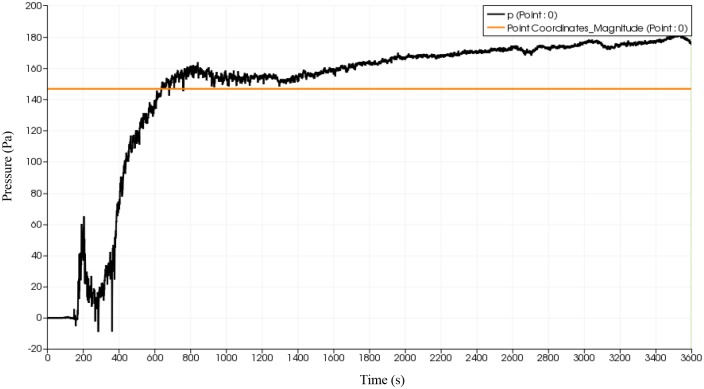
Graph of pressure versus time (perimeter) from simulation data.

Given that pressure could have detrimental effect in the deformation of the flip chip and solder balls, a probe is placed at the start of the inlet to monitor the fluid flow advancement at the onset of collision with solder bumps. The effect of reverse flow on the pressure distribution is also of interest. Using simulation, a probe is placed at approximately 10% filling percentage as illustrated in Fig 20. The same probe is place at the same location for all 3 types of orientation. The plot of pressure variation at the specified probe location over time are depicted in Figs 21, 22 and 23. All three orientations have same graph trend where the pressure is fluctuated at beginning of the fluid flow before 40 seconds and then reaches the steady state around 80 seconds. The continuous collisions between the particles in the fluid and the reverse flow lead to the creation of high and low pressures spots. Comparatively, BGA with perimeter and middle empty orientation reaches the steady state around 180 Pa whereas BGA with full orientation reaches the steady state around 190 Pa. It can be say that as the number of solder balls are increased with more reverse flow occurring at each ball, the pressure of the flow also increases steadily.

### Velocity distribution of fluid flow

**Fig 24:**
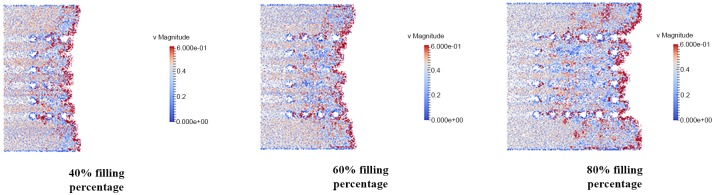
Velocity distribution of fluid flow (Orientation-perimeter).

**Fig 25:**
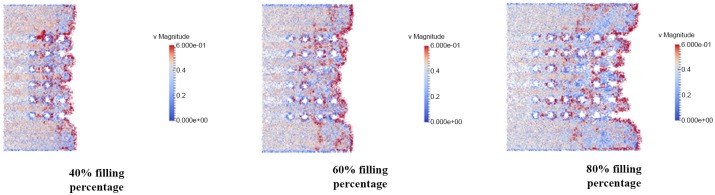
Velocity distribution of fluid flow (Orientation-middle empty).

**Fig 26:**
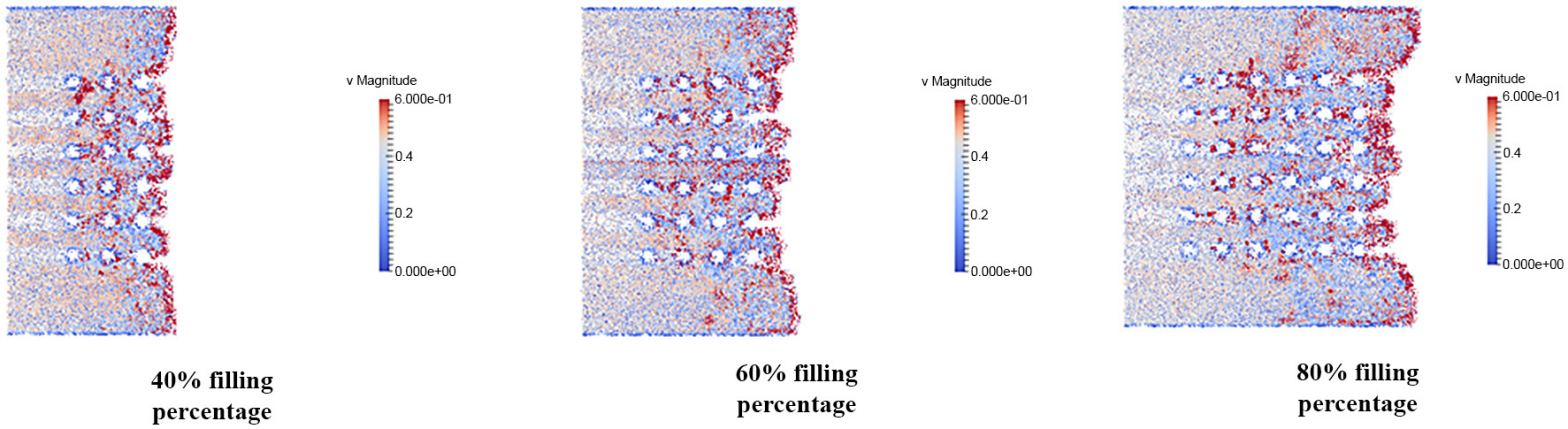
Velocity distribution of fluid flow (Orientation-full).

The velocity distribution is observed in filling percentage of 40%, 60% and 80% for all orientation (perimeter, middle empty and full) which shown in Figs 24, 25 and 26. The flow front of fluid flow has the highest velocity as indicated by red color for all 3 different orientations as illustrated in Figs 24, 25 and 26. This is due to the velocity of fluid before the flow front has reached its equilibrium with slight bounce back between fluid molecules. The high velocity fluid has high potential in damaging the solder bumps. Closer look at the vicinity of solder bump, it was clear that the velocity around the solder bumps retain its high velocity value. The phenomena is caused by consistent collision between the incoming fluid and the reflected fluid from near the solder bumps. This occurrence may force the solder balls sideways and in time lead to solder bump breakage. In addition, the non-uniform fluid velocity distribution throughout BGA surface is due to unbalance fluid flow as a result of slightly slower fluid flow in the middle compared to fluid flow rate at the edge.

### LBM and FVM comparison

**Fig 27:**
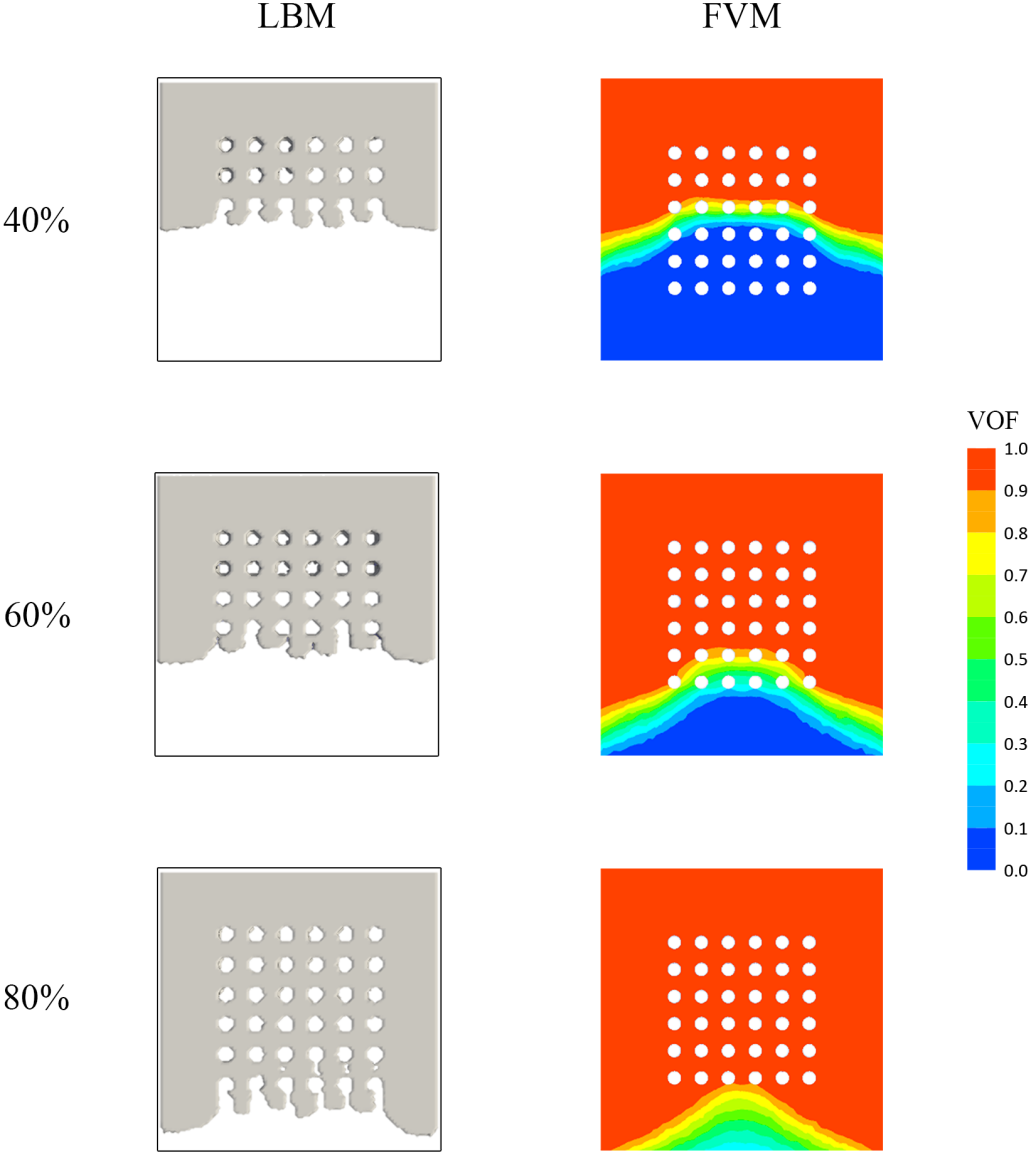
Percentage of volume of fraction (VOF) contour at different filling time for full orientation [37].

Current, most of the analysis relating to encapsulation process are conducted using FVM based software. Though FVM provide an accurate and reliable simulation in comparison to experimental findings [36] detection of micro-defect, i.e. void formation is still relatively difficult to be visualized during the encapsulation process. This voids if not detected earlier could lead to oxygen to be trapped in the voids. Subsequently, if the package is heated up to certain temperature, the oxygen could ignite leading to “pop corning” effect and package cracking [2]. Fig 27 compares the simulation results obtained using FVM compared to LBM based formulation for different filling percentage. In terms of the mold flow propagation, both LBM and FVM formulation gave good side by side free surface movement. This agreement shows good potential of LBM in modelling free surface movement compared to the conventional FVM formulation. The detection of void formation however, is not visible in FVM based software. As depicted in Fig 27, LBM formulation managed to capture the formation of void during the encapsulation process. At 40% and 60% filling percentage, the formation of voids are mostly located at the vicinity of the solder ball due to reverse flow phenomena [35,37]. At 80% filling percentage, the problem of voids formation is more severe and is concentrated at final two rows of the solder balls. In the study by C. S. Lau, the voids formation can be detected by FVM based formulation however, it was seen located only at regions near solder balls. The ability of LBM to simulate the voids formation is attributed to its ability to effectively model and capture multiphase free-surface flow. The voids formation typically occur as a results of interaction between air particles and mold particles. These interactions typically occur at micro-scale level is accounted accurately in the LBM formulation. For this reason the voids can be captured and visualized clearly.

It should be noted that FVM seems to capture the retention of the flow front surface much better with relatively less viscous fingering compared to the LBM formulation. From Fig 27, it can be seen that the formation of viscous fingering is apparent in the LBM simulation result as opposed to the FVM simulation result. Viscous fingering is the uneven, finger-like profile formed at the interface of two different fluids due to the difference in viscosity of the fluids. The reason viscous fingering is apparent in LBM simulation but not in FVM simulation is partly due to the input parameters used in the FVM simulation, specifically the wall adhesion angle. The pre-set wall adhesion angle is dependent on both the encapsulant material and also the PCB itself and such, further experiments are required to accurately determine this contact angle value. This contact angle value however is not set in the LBM formulation and the viscous fingering is only dependent on the surface tension value as well as the bounce back boundary condition that occurs during fluid to wall contact. In addition, the relatively finer detail due to the particle nature of the LBM formulation, could have contributed to this viscous fingering of the flow front profile. The same argument can used to explain the detection of voids formation that are only visible in the LBM based solver [35]. This viscous fingering however, has minor effect on the filling time, pressure and velocity profile of the flow front.

## Conclusion

This study discloses the importance of the solder bump arrangement in fluid flow filling time, pressure distribution and velocity distribution on the ball grid array (BGA). A 3-dimensional simulation model with the dimension of 72mm × 38mm × 15mm is generated using volume of fluid (VOF) method in order to duplicate the fluid flow phenomenon in capillary underfilling process. The fluid flow simulation is validated by the experiment and it was proven that LBM is capable of predicting the fluid flow motion during the encapsulation process. Both simulation and experimental findings show that perimeter orientation has fastest flow followed by BGA with middle empty and full orientations. This is due to relatively less number of solder bumps in BGA with perimeter orientation which hinders the fluid flow. The study also shows non-uniform pressure distribution throughout the encapsulation domain with lower flow rate at middle portion as a results of obstruction by the solder bumps. BGA with perimeter orientation has a higher pressure fluid at the middle of BGA surface compared to the middle empty and full orientations because of less solder bumps support. Less solder bumps support in the middle of BGA surface can cause downward deformation of BGA and reduce gap of fluid flow which induce high pressure at the middle portion. In addition, fluid inlet has high pressure distribution for all 3 different orientations as result of impact created by the incoming fluid from the inlet and reflected fluid from the solder bump. There are high velocity distribution around the solder bump also due to collision between the incoming fluid and reflected fluid from the solder bump. High pressure and velocity of fluid flow will causes unintended defects such as solder bump damage and chip deformation. This paper reveals the significance of solder bumps arrangement in affecting the pressure and velocity distribution which lead to the packaging reliability problems. It was shown that the increase in number of solder bump count reduces the pressure and velocity of the fluid flow. In terms of comparison between FVM and LBM formulation, good agreement can be formed for the mold flow propagation. However, only LBM formulation managed to capture the formation of voids. Consequently, this yields an increase in reliability of the electronic package. This study also shows the capability of Palabos software in generating fluid flow simulation models. High amount of detail offered by LBM allows accurate prediction of fluid flow problems. Moving forwards, LBM can be seen as a viable alternative to other conventional software to solve microscale electronic packaging problems.

## Supplementary Data

The figures available in this paper are available online and can be downloaded from Figshare database according to reference [38].
